# Correction: Christov, I.P. Effects of Spatial Nonlocality versus Nonlocal Causality for Bound Electrons in External Fields. *Entropy* 2022, *24*, 840

**DOI:** 10.3390/e24111521

**Published:** 2022-10-25

**Authors:** Ivan P. Christov

**Affiliations:** 1 Physics Department, Sofia University, 1164 Sofia, Bulgaria; ivan.christov@phys.uni-sofia.bg; 2 Institute of Electronics, Bulgarian Academy of Sciences, 1784 Sofia, Bulgaria

The author would like to make the following corrections to the published paper [1]: The time units in the titles of Figure 2 and Figure 3 are in femtoseconds (fs) and not in atomic units (a.u.). The update to the figures does not in any way affect the scientific conclusions of the published article. The corrected figures are as follows:

## Figures and Tables

**Figure 2:**
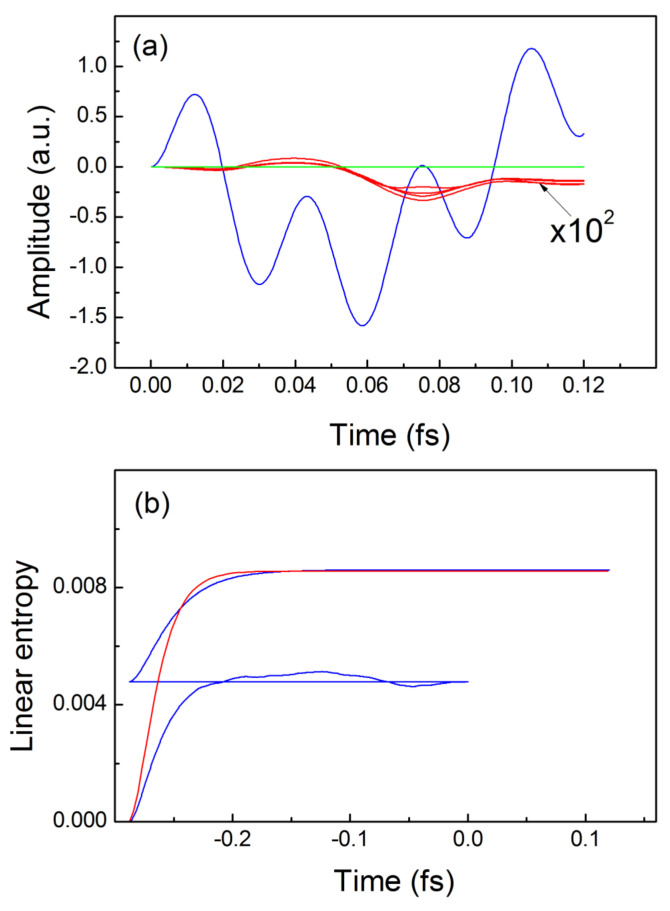
Trajectories and linear quantum entropies for non-interacting electrons: (**a**) exact trajectories for the driven electron—blue line, for the idler electron—red lines, TDQMC result—green line; (**b**)—buildup and real-time dependence of the exact linear entropy—red line, and from the TDQMC calculation—blue line.

**Figure 3:**
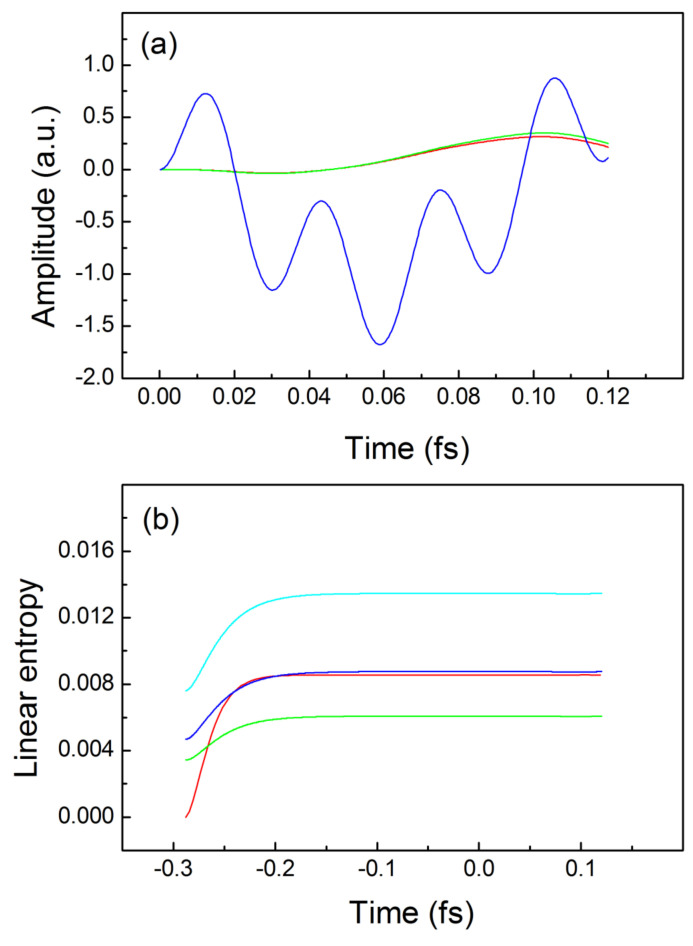
Trajectories and linear quantum entropies for interacting electrons: (**a**) exact trajectories for the driven electron—blue line, for the idler electron—red line, TDQMC result—green line; (**b**) buildup and real-time dependence of the exact linear entropy—red line, and the TDQMC result for: σ=0.82a.u.—blue line, σ=0.70a.u.—light-blue line, σ=1a.u.—green line. In (**b**), both electrons are driven by the external field.
